# Apes perform like infants in false-belief tasks

**DOI:** 10.3758/s13420-017-0268-z

**Published:** 2017-04-07

**Authors:** Thomas Bugnyar

**Affiliations:** Department of Cognitive Biology, University of Vienna, Vienna, Austria

**Keywords:** Theory of mind, False beliefs, Great apes

## Abstract

Although the extent to which some nonhuman animals understand mental states is currently under debate, attributing false beliefs has been considered to be beyond their limits. A recent study by Krupenye, Kano, Hirata, Call, and Tomasello (*Science, 354*, 110–114, 2016) shows that great apes pass a false-belief task when they are tested with an anticipatory-looking paradigm developed for nonverbal human infants.

Humans are renowned for operating with a “theory of mind.” At some point during development, we come to understand that other humans are intentional beings whose behavior is driven by mental states such as perceptions, beliefs, or knowledge. We easily generalize our ability to nonhuman animals and predict their behavior on the basis of what they may see, know, or want. We also commonly assume that such an attribution is a typical human ability, which exceeds the cognitive realm of (most) nonhuman animals. Although hardly anyone questions that attributing mental states is cognitively sophisticated, intense debates exist about whether this ability is genuinely unique to humans or may also be found in some nonhuman animals (Heyes, 2015).

Recent conceptual and methodological advancements have shifted the focus of discussion away from the question of whether or not some animals might possess a theory of mind to the question of which aspects of theory of mind can be found in those animals (e.g., Call & Tomasello, 2008). A series of behavioral experiments on chimpanzees produced converging evidence that these apes can predict others’ behavior on the basis of an understanding of the others’ goals, perceptions, and knowledge states in the context of food competition. Similar findings were reported for Rhesus macaques, suggesting that precursors of a human theory of mind can be found in Old World monkeys. Perhaps even more surprisingly, attribution of perception, knowledge, and desire states has also been reported for food-caching birds such as ravens and scrub jays, suggesting that elements of a theory of mind might have evolved independently in distantly related taxonomic groups (Bugnyar, Reber, & Buckner, 2016). What is not yet clear, however, is whether any of those animals can represent others’ beliefs. From a cognitive point of view, belief attribution is particularly interesting, because it requires recognizing that others’ actions are driven not by reality but by beliefs about reality, even when those beliefs are false.

In human children, false-belief understanding develops around 4 years of age and is traditionally tested with versions of the “Sally-Ann” task—that is, a puppet play in which children must explicitly predict a mistaken puppet agent’s future actions. Modified versions of the test use a nonverbal design and a child’s spontaneous gaze response as a measure. The underlying assumption of this paradigm is that children have a tendency to look at a location in anticipation of an impending event—that is, what they predict an agent will do, even when this agent may hold a false belief about a situation. These studies have indicated that implicit knowledge about others’ beliefs is already present in 2-year-old infants (Southgate, Senju, & Csibra, 2007).

A recent study by Krupenye, Kano, Hirata, Call, and Tomasello (2016) used this anticipatory-looking paradigm to test for false-belief understanding in three species of apes. Chimpanzees, bonobos, and orangutans watched short videos on a monitor while their gaze was noninvasively recorded using an infrared eyetracker. The general design and procedure of the test was the same as in the study with infants (Southgate et al., 2007). The scenarios shown in the videos, however, were designed to be meaningful to apes and featured competitive interactions for status and for a specific item. In one scenario, an ape-like character (human dressed up as gorilla) hit a human trainer on the back and then hid itself in one of two haystacks; in the other scenario, the ape-like character stole an item previously hidden by the human trainer and hid it in a different location. In both scenarios, the trainer then searched for the goal object (ape-like character or the hidden item) in one of the two locations. The tested apes always saw three video sequences per scenario in a row. The first two sequences served as familiarization trials and established that the object (e.g., the ape-like character) could be hidden in either of the two locations, and that the human trainer would search for it in its true location. In the final test sequence, the human trainer was also present in the beginning and witnessed the initial hiding of the object, before he briefly left the scene. In contrast to the familiarization trials, the object was moved from one location to the other, and eventually was removed completely (e.g., the ape-like character moved from the left to the right haystack, and finally left the scene). The object’s movement between the locations could occur while the trainer was still present or while he was already absent, creating differences in the trainer’s beliefs about the object’s location; the object’s complete retrieval, however, was always done in the trainer’s absence, creating a false belief in the trainer about the object’s presence in either condition. Upon his return, the human trainer ambiguously approached the two locations (e.g., centrally between the two haystacks) without providing any directional cues. At this critical time point, the apes’ anticipatory looks were assessed on the basis of their first looks to the target location—that is, where the trainer would falsely believe the object to be, or the other location.

The majority of the apes indeed looked at the target location first—that is, they accurately anticipated the goal-directed behavior of the human trainer who held a false belief. This result is of high importance from conceptual and methodological points of view: It not only extends the list of mental states that apes have responded to in behavioral experiments, but for the first time indicates that attributing reality-incongruent mental states may be within the capacity of some nonhuman animals, at least on an implicit level. The study also shows what is possible when renowned primate labs in Europe, Japan, and the USA pool their expertise and resources. One of the practical outcomes of this collaboration was access to a fairly large sample size of around 40 apes, which was essential for applying a design developed for human infants and detecting subtle differences in visual behavior. In fact, about 1/4 of the subjects did not look at any of the locations in the short test situation, and thus had to be omitted from the analysis; for the remaining 29 subjects, a combined analysis of the two scenarios yielded a statistically reliable result. As was described above, the study features an excellent design implementing several controls and using state-of-the-art eyetracking technology, in much the same way as in studies on human infants; the design also pays attention to biological predispositions and the daily life experience of the apes, framing the test scenarios in an ecologically relevant context. Interestingly, no species difference could be detected, nor was there any significant difference between the two false-belief conditions (i.e., when the first movement of the object occurred: with the trainer being either still present or already absent). Possibly these results were affected by low statistical power, but the variation between species and/or conditions was small.

The critical point of this type of study, however, is that it is open to alternative, nonmental explanations, such as the learned rule “agents search for things where they last saw them.” The authors openly discuss this possibility and rightly point out that this critique applies to any implicit false-belief task that relies on a change of locations. They further argue that a rule-based explanation using domain-general mechanisms of attention, learning, and memory cannot account for the results obtained in previous theory-of-mind-related experiments on apes. This argumentation is intuitive, but it needs to be treated as a working hypothesis and tested against submental alternatives in future studies. The study presently discussed should spark a new focus on how this enterprise can be achieved.

Given that the methodological critique applies to both ape and infant studies, the authors rightly challenge the common view that the ability to pass an implicit false-belief task is specific to humans. Following the logic and interpretation of the infant studies, we may argue that three species of great apes show an implicit understanding of belief. From an evolutionary perspective, elements of belief attribution may thus go back to the last common ancestor between humans and orangutans. Hence, the study by Krupenye et al. (2016) not only sets new standards for studying false-belief attribution, but it represents a crucial step forward in our attempt to understand the evolutionary emergence of theory of mind.
